# Design and Preclinical Evaluation of a Novel Prostate-Specific Membrane Antigen Radioligand Modified with a Transthyretin Binder

**DOI:** 10.3390/cancers16071262

**Published:** 2024-03-23

**Authors:** Christian Vaccarin, Ana Katrina Mapanao, Luisa M. Deberle, Anna E. Becker, Francesca Borgna, Giovanni Marzaro, Roger Schibli, Cristina Müller

**Affiliations:** 1 Center for Radiopharmaceutical Sciences ETH-PSI, Paul Scherrer Institute, 5232 Villigen-PSI, Switzerland; christian.vaccarin@psi.ch (C.V.); ana.mapanao@psi.ch (A.K.M.); luisa.deberle@gmail.com (L.M.D.); beckeranna.e@gmail.com (A.E.B.); francesca.borgna.1@gmail.com (F.B.); roger.schibli@pharma.ethz.ch (R.S.); 2 Department of Pharmaceutical and Pharmacological Sciences, University of Padua, I-35131 Padua, Italy; giovanni.marzaro@unipd.it; 3 Department of Chemistry and Applied Biosciences, ETH Zurich, 8093 Zurich, Switzerland

**Keywords:** transthyretin binder, plasma protein binding, PSMA radioligand, prostate cancer, lutetium-177, PC-3 PIP tumor cells

## Abstract

**Simple Summary:**

Prostate-specific membrane antigen (PSMA)-targeting radioligands have been used clinically to treat metastatic prostate cancer. To deliver a sufficient radiation dose to eliminate cancer cells, the accumulation of the radioligand in the tumor should be high. Therefore, small-molecular-weight molecules have been modified with albumin binders to enhance their blood circulation time and, hence, increase the tumor uptake. The goal of this study was to investigate the utility of a transthyretin binder (TB-01) to achieve high tumor uptake of the resultant PSMA radioligand. Indeed, the newly developed [^177^Lu]Lu-PSMA-TB-01 demonstrated enhanced blood circulation as compared to the clinically employed [^177^Lu]Lu-PSMA-617. Consequently, a significantly higher accumulation of activity was observed in the tumor tissue, while activity retention in off-target tissue was only higher in the kidneys, but otherwise in the same range as for [^177^Lu]Lu-PSMA-617.

**Abstract:**

Transthyretin binders have previously been used to improve the pharmacokinetic properties of small-molecule drug conjugates and could, thus, be utilized for radiopharmaceuticals as an alternative to the widely explored “albumin binder concept”. In this study, a novel PSMA ligand modified with a transthyretin-binding entity (TB-01) was synthesized and labeled with lutetium-177 to obtain [^177^Lu]Lu-PSMA-TB-01. A high and specific uptake of [^177^Lu]Lu-PSMA-TB-01 was found in PSMA-positive PC-3 PIP cells (69 ± 3% after 4 h incubation), while uptake in PSMA-negative PC-3 flu cells was negligible (<1%). In vitro binding studies showed a 174-fold stronger affinity of [^177^Lu]Lu-PSMA-TB-01 to transthyretin than to human serum albumin. Biodistribution studies in PC-3 PIP/flu tumor-bearing mice confirmed the enhanced blood retention of [^177^Lu]Lu-PSMA-TB-01 (16 ± 1% IA/g at 1 h p.i.), which translated to a high tumor uptake (69 ± 13% IA/g at 4 h p.i.) with only slow wash-out over time (31 ± 8% IA/g at 96 h p.i.), while accumulation in the PC-3 flu tumor and non-targeted normal tissue was reasonably low. Further optimization of the radioligand design would be necessary to fine-tune the biodistribution and enable its use for therapeutic purposes. This study was the first of this kind and could motivate the use of the “transthyretin binder concept” for the development of future radiopharmaceuticals.

## 1. Introduction

Radionuclide therapy based on tumor-targeting small molecules and peptides evolved as an effective concept to treat metastasized cancer [1]. Unlike larger proteins and antibodies, the manufacturing of small-molecular-weight molecules using conventional chemistry methodologies is feasible at reasonable cost [2]. In the future, medium- or high-throughput screening techniques such as phage display [3] or DNA-encoded chemical libraries [4] may offer fast identification of novel small molecules that could be used as vehicles for the efficient delivery of radionuclides to the tumor site. The number of currently used radiopharmaceuticals for nuclear imaging in clinical practice is relatively large; however, only a small number of drugs have been used for targeted radionuclide therapy. [^177^Lu]Lu-DOTATATE (Lutathera^TM^ [5]) was approved by the authorities for the treatment of gastroenteropancreatic neuroendocrine tumors [6], and [^177^Lu]Lu-PSMA-617 (Pluvicto™ [7]) is a registered drug for the treatment of metastatic castration-resistant prostate cancer [8].

Despite the large number of advantages presented by small molecules, their overly fast blood and kidney clearance limits their accumulation in the malignant tissue. Consequently, large quantities of activity must be injected to reach therapeutically effective tumor doses. The design and optimization of new small-molecular-weight radiopharmaceuticals have, therefore, been a subject of continuous research. The aim has always been to improve the radiopharmaceutical’s pharmacokinetic properties and, hence, improve therapeutic efficacy [9].

The functionalization of radiopharmaceuticals with albumin-binding entities (the *p*-iodophenyl entity, truncated Evans blue, etc.) to enhance the serum half-life and, therewith, increase the tumor uptake has been investigated extensively in recent years [9]. Preclinical studies have demonstrated that strong albumin-binding of the radiopharmaceutical may be advantageous in some cases [10], but in other cases, moderate albumin binding led to a more favorable tissue distribution profile of the respective radioligand [11,12,13]. An extensive blood circulation of the radiopharmaceutical leads to an increased bone marrow dose, which may become dose-limiting. As an example, albumin binders with moderate albumin-binding affinity such as *p*-chlorophenyl [13] and the *p*-tolyl entities [11] as well as ibuprofen [12] conferred more favorable properties of the resultant PSMA radioligands than the strong *p*-iodophenyl entity [11,14,15] and truncated Evans Blue [16]. It is, thus, noteworthy that the same albumin binder may not be the most favorable for each tumor-targeting agent [10,11].

These observations highlight the importance of fine-tuning the plasma protein-binding properties of radiopharmaceuticals and the need for a multitude of building blocks available to combine the most suitable entity with a particular tumor-targeting agent. In the present study, we exemplified the modification of a PSMA ligand with a transthyretin-binding entity based on AG10, which was previously developed by Penchala et al. [17].

Transthyretin is a tetrameric plasma protein of a size (~55 kDa) comparable to serum albumin; however, it is present in the blood at a considerably lower concentration (~5 μM vs. ~800 μM for human serum albumin). It has a half-life time of ~2 days in human blood plasma, which is considerably shorter than that of serum albumin (~19 days) [18]. The physiological function of transthyretin mainly refers to the transport of thyroid hormones (e.g., thyroxine) and holo-retinol binding protein (i.e., with bound retinol) [19]. By combining the concepts of transthyretin-binding therapeutics with an active targeting strategy, a series of PSMA-binding drug conjugates were previously developed utilizing the AG10-based transthyretin binder [20,21]. This strategy improved the pharmacokinetic properties, and, hence, the therapeutic efficacy of the drug conjugate was enhanced compared to the non-targeted payload as well as compared to the PSMA-targeted chemotherapeutic lacking a transthyretin binder [20,21].

In the present study, the PSMA-617 scaffold was chosen for derivatization using the AG10-based transthyretin binder (herein referred to as TB-01) as a model construct to demonstrate the usefulness of transthyretin-binding properties of small-molecular-weight radiopharmaceuticals. The resultant radioligand, [^177^Lu]Lu-PSMA-TB-01, was tested in vitro and in vivo to explore its transthyretin-binding properties and related benefits to enhance the accumulation in the tumor tissue.

## 2. Materials and Methods

### 2.1. Synthesis of PSMA-TB-01

**Scheme 1:**
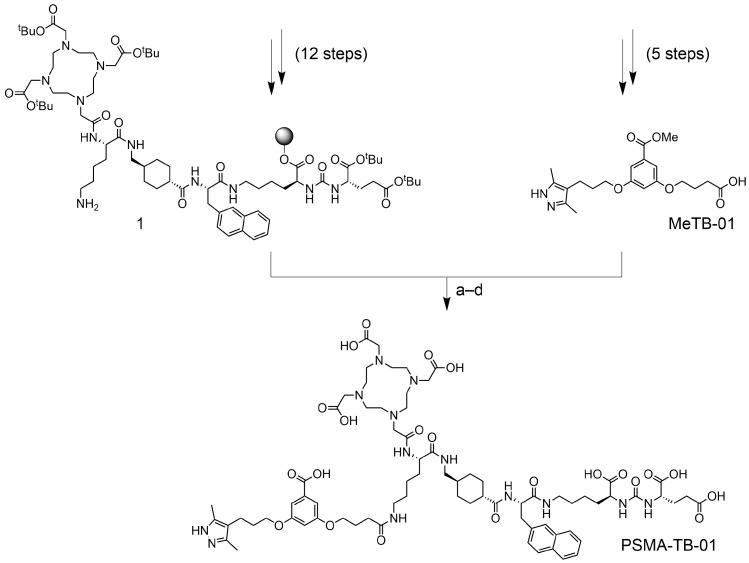
Synthesis scheme for the preparation of PSMA-TB-01. Reaction conditions: (a) HBTU, DIPEA, DMF, RT, 2 × 1 h; (b) HFIP, DCM, RT, 2 × 30 min; (c) TFA, TIS, Milli-Q water, RT, 4.5 h; (d) LiOH, Milli-Q water, MeCN, RT, 1 h.

The PSMA ligand backbone with a lysine residue integrated for derivatization with an additional entity (compound **1**) was synthesized using the method of solid-phase peptide synthesis (SPPS) as previously reported (Supplementary Materials, Scheme S1) [11]. The transthyretin-binding entity was synthesized as a protected intermediate (MeTB-01) using classic organic chemistry in solution as previously reported for the preparation of DOTA-TB-01 (Supplementary Materials, Scheme S2) [22]. Subsequently, the free carboxylic function of MeTB-01 was conjugated with the primary amine of the resin-immobilized PSMA ligand precursor (**1**) (Scheme 1). Cleavage from the resin and removal of the *tert*-butyl- and methyl-ester-protecting groups yielded the crude PSMA-TB-01. The substance was purified using semipreparative HPLC to obtain PSMA-TB-01 (Supplementary Materials).

### 2.2. Preparation and Characterization of the Radioligand

The radiolabeling of PSMA-TB-01 with lutetium-177 (no-carrier added [^177^Lu]LuCl_3_ in 0.04 M HCl; ITM Medical Isotopes GmbH, Munich, Germany) was performed under standard labeling conditions at pH 4.5. The assessment of the radiolytic stability and distribution coefficient (logD) of [^177^Lu]Lu-PSMA-TB-01 was performed according to previously published protocols (Supplementary Materials) [11,14].

### 2.3. Cell Culture

PSMA-positive PC-3 PIP and PSMA-negative PC-3 flu tumor cells were obtained from Prof. Martin Pomper (Johns Hopkins University School of Medicine, Baltimore, MD, USA) [23,24]. The cells were cultured in RPMI-1640 cell culture medium supplemented with 10% fetal calf serum, l-glutamine, antibiotics, and puromycin (2 µg/mL) and split twice a week using PBS/EDTA and trypsin for detachment.

### 2.4. Cell Uptake and PSMA-Binding Affinity

Cell uptake and internalization studies of [^177^Lu]Lu-PSMA-TB-01 (50 MBq/nmol) were performed using PC-3 PIP and PC-3 flu cells, following a slight modification of a previously reported protocol (Supplementary Materials) [25]. The PSMA-binding affinity was determined with PC-3 PIP cells using [^177^Lu]Lu-PSMA-TB-01 (5 MBq/nmol) to obtain the K_D_ values as previously reported (Supplementary Materials) [25]. All in vitro studies were performed three times in triplicate and reported as the average (±standard deviation, SD) of the individually performed experiments.

### 2.5. Binding of the Radioligand to Human and Mouse Plasma

The binding of [^177^Lu]Lu-PSMA-TB-01 (50 MBq/nmol) to proteins in undiluted human blood plasma (Stiftung Blutspende SRK Aargau-Solothurn, Aarau, Switzerland) and murine blood plasma (Rockland Immunochemicals, Inc., Pottstown, PA, USA, Lot N° 32321) was determined using an ultrafiltration assay as previously reported [26]. Briefly, after incubating the radioligand with blood plasma at 37 °C for 30 min, the samples were placed on ice. After the addition of 150 µL ice-cold PBS, the suspensions were transferred to Amicon centrifugal filters (cut-off of 10 kDa; Merck Millipore, Burlington, MA, USA). The free radioligand (not bound to proteins) was separated through centrifugation at 14,000 rcf at 4 °C for 30 min. The protein-bound fraction of the radioligand was collected by placing the filter insert inverted in another tube followed by centrifugation at 200 rcf for 3 min. The activity of the samples was measured in a γ-counter (PerkinElmer, Waltham, MA, USA, Wallac Wizard 1480), and it is expressed as a percentage of the total recovered activity.

### 2.6. Evaluation of the Affinity of [^177^Lu]Lu-PSMA-TB-01 to Serum Proteins

Dilutions of human serum albumin (HSA, Sigma, St. Louis, MO, USA, A3782, Lot N° SLBZ2785) in the concentration range of 0.070 µM to 700 µM as well as dilutions of human transthyretin (Sigma, P1742, Lot N° SLCL2282) in the concentration range of 5 pM to 70 µM were prepared in PBS. Human blood plasma (Stiftung Blutspende SRK Aargau-Solothurn, Switzerland) was diluted in PBS at a concentration range of 0.43 nM to 5 µM, calculated based on transthyretin content which is reported to be 5 µM in undiluted human blood plasma [27]. A fixed amount of [^177^Lu]Lu-PSMA-TB-01 (0.3 MBq, 6 pmol, 15 µL) was added to all samples followed by performing the ultrafiltration procedure as reported in Section 2.5. The experiments were performed 3 times, and the values are presented as the average (±SD). The percentage of protein-bound [^177^Lu]Lu-PSMA-TB-01 was plotted against the protein-to-radioligand molar concentration ratios on a logarithmic scale. A non-linear regression curve was fitted to the data using GraphPad Prism (version 8). The relative binding affinity was calculated as the inverse half-maximum binding (B_50_) of the radioligand to compare its binding to transthyretin, HSA, and plasma proteins in human blood plasma. The 1/B_50_ value obtained for [^177^Lu]Lu-PSMA-TB-01 bound to transthyretin was set at 1.0 (Supplementary Materials).

### 2.7. Blocking of the Plasma Protein Binding of [^177^Lu]Lu-PSMA-TB-01

The binding of [^177^Lu]Lu-PSMA-TB-01 to plasma proteins diluted at approximately 1:10 *(v/v)* of their respective physiological concentrations was evaluated in the presence of various blocking agents. Transthyretin (0.5 μM), HSA (70 μM), and human blood plasma (transthyretin concentration ~0.5 µM) were incubated with thyroxine (50 µM), TB-01 (50 µM), ibuprofen (7 mM), or warfarin (7 mM) for 15 min at 37 °C. After the addition of a fixed amount of [^177^Lu]Lu-PSMA-TB-01 (0.3 MBq, 6 pmol, 15 µL), the samples were incubated again for 30 min at 37 °C. The amount of protein-bound radioligand was determined by an ultrafiltration procedure as in Section 2.5. (Supplementary Materials).

### 2.8. In Vivo Studies

All applicable international, national, and/or institutional guidelines for the care and use of animals were followed. In particular, all animal experiments were carried out according to the guidelines of the Swiss Regulations for Animal Welfare. The preclinical studies were ethically approved by the Cantonal Committee of Animal Experimentation and permitted by the responsible cantonal authorities (License N° 75668 and related extensions). Female BALB/c nude mice were purchased from Charles River Laboratories (Sulzfeld, Germany) at the age of 5–6 weeks and acclimatized after arrival at PSI for at least one week.

### 2.9. Biodistribution Studies

BALB/c nude mice were subcutaneously inoculated with PSMA-positive PC-3 PIP cells (6 × 10^6^ cells in 100 μL Hank’s balanced salt solution (HBSS)) on the right shoulder and with PSMA-negative PC-3 flu cells (5 × 10^6^ cells in 100 μL HBSS) on the left shoulder as previously reported [11,14]. Approximately two weeks later, when the xenografts reached an approximate volume of ~100–200 mm^3^, the mice were intravenously injected with [^177^Lu]Lu-PSMA-TB-01 (5 MBq/nmol, 1 nmol; diluted in saline with 0.05% bovine serum albumin, BSA). Mice (*n* = 4) were sacrificed at 1 h, 4 h, 24 h, and 96 h post injection (p.i.). Collected tissues and organs were weighed and counted for activity using a γ-counter (PerkinElmer Wallac Wizard 1480). The data are expressed as the percentage injected activity per gram of tissue mass (% IA/g) using standards of the injected activity measured at the same time to obtain decay-corrected values.

### 2.10. SPECT/CT Imaging Studies

SPECT/CT experiments were performed using a dedicated small-animal SPECT/CT scanner (NanoSPECT/CT^TM^, Mediso Medical Imaging Systems, Budapest, Hungary) as previously reported [14]. SPECT/CT scans of the PC-3 PIP/PC-3 flu tumor-bearing mice (*n* = 2) were acquired at 4 h, 24 h, and 48 h after intravenous injection of [^177^Lu]Lu-PSMA-TB-01 (25 MBq, 1 nmol, 100 μL saline containing 0.05% BSA). The SPECT/CT image acquisition of mice lasted approximately 60 min. Inhalation anesthesia using a mixture of isoflurane and oxygen was applied to immobilize mice while scanning. Real-time CT reconstruction used a cone-beam-filtered back-projection and reconstruction of SPECT data was performed using the HiSPECT software (version 1.4.3049, Scivis GmbH, Göttingen, Germany). The data were post-processed using VivoQuant (version 3.5, inviCRO Imaging Services and Software, Boston, MA, USA). A Gaussian post-reconstruction filter (full-width at half-maximum = 1.0 mm) was applied and the scale of activity accumulation was set as indicated on the images (minimum value: 0.5 Bq/voxel; maximum value: 50 Bq/voxel).

## 3. Results

### 3.1. Synthesis of PSMA-TB-01

The synthesis intermediate (compound **1**) was obtained using an established SPPS methodology. The chemical identity of the resin-immobilized compound was confirmed by cleaving a small portion of the resin with trifluoroacetic acid (TFA) and by subsequent analysis of the supernatant using liquid chromatography–mass spectrometry (LC-MS) (Supplementary Materials). The ^1^H-NMR spectra of the synthesis intermediate and MeTB-01 correlated well with the previously reported spectra (Supplementary Materials) [22]. The chemical identity of MeTB-01 was further confirmed by high-resolution mass spectrometry (HRMS) analysis (HRMS (ESI) of C_20_H_25_N_2_O_6_: *m*/*z*_calc_ [M−H]^−^ = 389.1718; *m*/*z*_meas_ [M−H]^−^ = 389.1695).

After the final coupling step, global deprotection, and HPLC purification, PSMA-TB-01 was obtained in an overall yield of 2%. The chemical purity determined by LC-MS analysis was >98%. The identity of PSMA-TB-01 was confirmed using HRMS (HRMS (ESI) of C_74_H_105_N_13_O_22_: *m*/*z*_calc_ [M + H]^+^ = 1528.7575; *m*/*z*_meas_ [M + H]^+^ = 1528.7562).

### 3.2. Preparation of [^177^Lu]Lu-PSMA-TB-01

Radiolabeling of PSMA-TB-01 with lutetium-177 was readily achieved at a molar activity of 50 MBq/nmol with a radiochemical purity of >98% (Supplementary Materials, Figure S1). [^177^Lu]Lu-PSMA-TB-01 was stable in PBS over 24 h at a high activity concentration (500 MBq/mL) demonstrated by >99% intact radioligand (*n* = 3) determined at this timepoint. The *n*-octanol/PBS distribution coefficient (logD value) of [^177^Lu]Lu-PSMA-TB-01 was −3.4 ± 0.1.

### 3.3. Cell Uptake and PSMA-Binding Affinity

**Figure 1:**
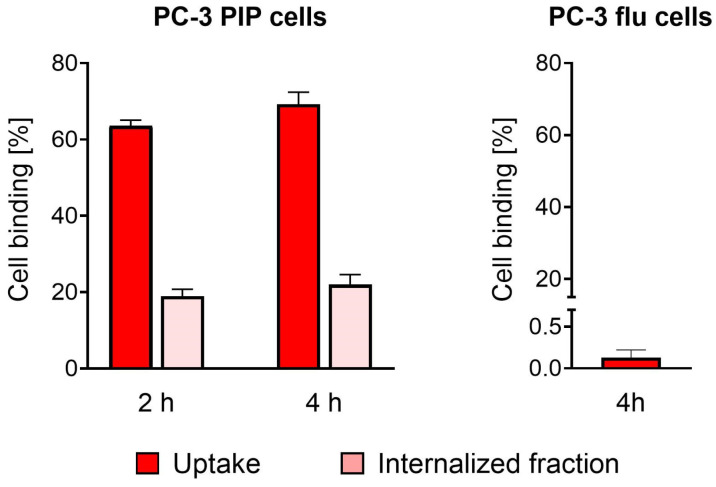
Uptake and internalization of [^177^Lu]Lu-PSMA-TB-01 in PSMA-positive PC-3 PIP cells. Negligible uptake of [^177^Lu]Lu-PSMA-TB-01 in PSMA-negative PC-3 flu cells.

The uptake of [^177^Lu]Lu-PSMA-TB-01 in PSMA-positive PC-3 PIP cells was 64 ± 1% after an incubation period of 2 h and reached 69 ± 3% after 4 h of incubation. The internalized fraction accounted for 19 ± 2% and 22 ± 3% of the total added activity after incubation for 2 h and 4 h, respectively. Negligible cell uptake (<0.5%) was seen in PSMA-negative PC-3 flu cells (Figure 1). The PSMA-binding affinity of [^177^Lu]Lu-PSMA-TB-01 was in the nanomolar range (K_D_ value of 23 ± 1 nM).

### 3.4. Plasma Protein-Binding Properties

**Figure 2:**
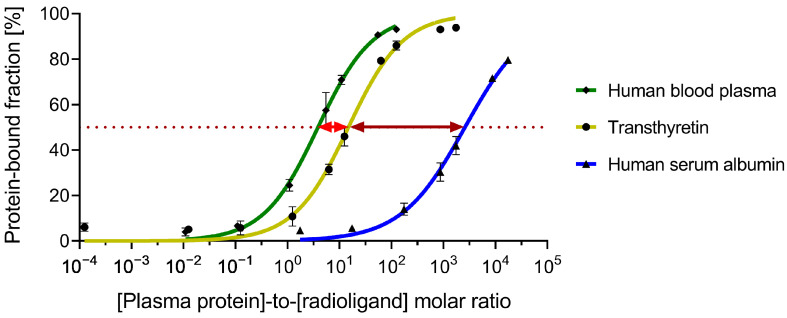
Fraction of the radioligand bound to variable concentrations of proteins in human blood plasma (calculated based on the transthyretin content), transthyretin, and human serum albumin (HSA). The B_50_ values differ by a factor of 174 for the binding of the radioligand to transthyretin and human serum albumin (indicated with a dark red arrow) and by a factor 3.9 for the binding of the radioligand to transthyretin and to the blood plasma proteins (indicated with a light red arrow). Red-dotted line: protein-bound fraction corresponding to 50%.

The plasma protein-binding curves at variable protein-to-radioligand molar concentration ratios revealed an ~174-fold higher affinity of [^177^Lu]Lu-PSMA-TB-01 to transthyretin (relative affinity set as 1.0) than to HSA (relative affinity: 0.006). The relative affinity to plasma proteins in human plasma (HSA and transthyretin) was 3.9-fold stronger (relative affinity: 3.9), calculated based on the transthyretin concentration. One should note the fact that HSA is present in human blood plasma dilutions in 140-fold higher concentrations than transthyretin (Figure 2, Supplementary Materials, Table S1).

When tested at physiological concentrations of the respective proteins, the fraction of [^177^Lu]Lu-PSMA-TB-01 bound to transthyretin was slightly higher (86 ± 2%) than the fraction bound to isolated HSA (80 ± 1%). Incubation with undiluted human and murine blood plasma resulted in 93 ± 1% and 91 ± 1% binding of the radioligand to plasma proteins, respectively.

**Figure 3:**
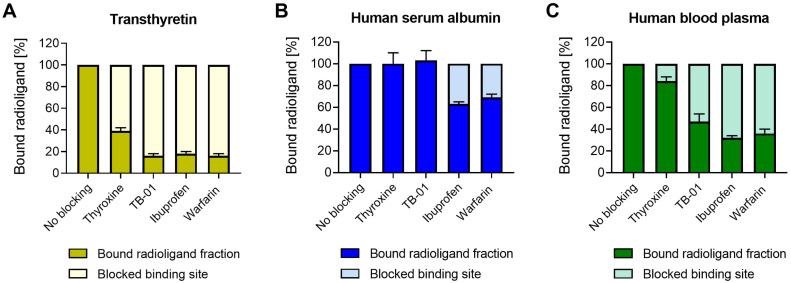
Relative amount of radioligand bound to diluted plasma proteins using various blocking agents. (**A**) Transthyretin (TTR, 0.5 µM), (**B**) human serum albumin (HSA, 70 µM), and (**C**) human blood plasma (corresponding to 0.5 µM TTR). Blocking agents were used at various concentrations: thyroxine (50 µM), TB-01 (50 µM), ibuprofen (7 mM), and warfarin (7 mM).

[^177^Lu]Lu-PSMA-TB-01 binding to isolated proteins (transthyretin and HSA) or proteins in human blood plasma was measured after dilution to 1:10 (*v/v*) of the physiological protein concentrations and after subsequent co-incubation with various blocking agents (Figure 3). The addition of thyroxine or TB-01 reduced the amount of transthyretin-bound [^177^Lu]Lu-PSMA-TB-01 to 39 ± 3% and 16 ± 2%, respectively, relative to the sample without the blocking solution. [^177^Lu]Lu-PSMA-TB-01 binding to transthyretin was also reduced to <20% upon co-incubation with ibuprofen and warfarin (Figure 3A). Studies performed in HSA revealed no blocking effects with the addition of thyroxine or TB-01, demonstrated by the fact that 100% of the radioligand was still bound to plasma proteins in HSA. In contrast, the amount of plasma protein-bound [^177^Lu]Lu-PSMA-TB-01 in HSA was reduced to 63 ± 2% and 69 ± 1% upon co-incubation with the known albumin binders ibuprofen and warfarin, respectively (Figure 3B). As expected, all the investigated blocking agents reduced the amount of protein-bound radioligand considerably in human plasma (~70 µM HSA and ~0.5 µM transthyretin content), where both transthyretin and HSA are present (Figure 3C).

### 3.5. Biodistribution of the Radioligand in Tumor-Bearing Nude Mice

**Figure 4:**
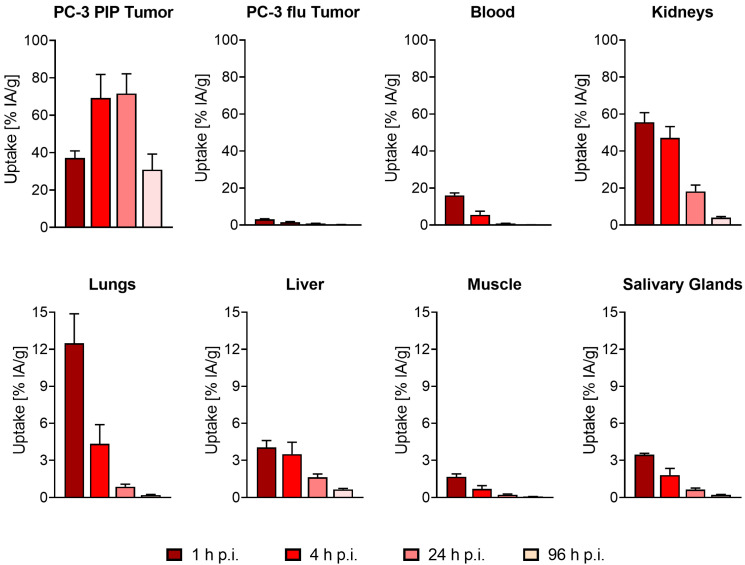
Time-dependent tissue distribution of [^177^Lu]Lu-PSMA-TB-01 in mice bearing PC-3 PIP and PC-3 flu tumors. The bars represent decay-corrected average (±SD) uptake values in the PC-3 PIP tumor, PC-3 flu tumors, blood, kidneys, lungs, liver, muscle, and salivary glands at 1 h, 4 h, 24 h, and 96 h after injection of the radioligand (*n* = 4 mice per timepoint).

The tissue distribution profile of [^177^Lu]Lu-PSMA-TB-01 was determined over 96 h after injection of the radioligand (Figure 4; Supplementary Materials, Table S2). Initially, blood activity was relatively high (16 ± 1% IA/g at 1 h p.i.) but cleared effectively over the first 24 h to <1% IA/g. As a result, a substantial accumulation of [^177^Lu]Lu-PSMA-TB-01 was seen in the PC-3 PIP tumor xenografts already at 1 h p.i. (37 ± 4% IA/g), which was further increased over the following hours to 69 ± 13% IA/g at 4 h after injection. The activity in the tumor tissue was retained well (72 ± 11% IA/g, at 24 h p.i.) and decreased only slowly over the following days (31 ± 8% IA/g, at 96 h p.i.). In the PSMA-negative PC-3 flu tumor xenograft, only low activity was detected (3.1 ± 0.3% IA/g at 1 h p.i.), which was not retained (0.81 ± 0.12% IA/g, at 24 h p.i.). Initial activity in the kidney was high (56 ± 5% IA/g at 1 h p.i.) and the clearance of [^177^Lu]Lu-PSMA-TB-01 was only slow (47 ± 6% IA/g at 4 h p.i., 18 ± 3% IA/g at 24 h p.i., and 4 ± 1% IA/g at 96 h p.i.). Low levels of activity were also observed in other organs and tissues with the highest levels in the lungs (12 ± 2% IA/g at 1 h p.i.), in the spleen (4.3 ± 0.2% IA/g at 1 h p.i.), and in the liver (4.0 ± 0.6% IA/g at 1 h p.i.); however, it decreased rapidly to <2% IA/g over the first 24 h after injection.

### 3.6. SPECT/CT Imaging

**Figure 5:**
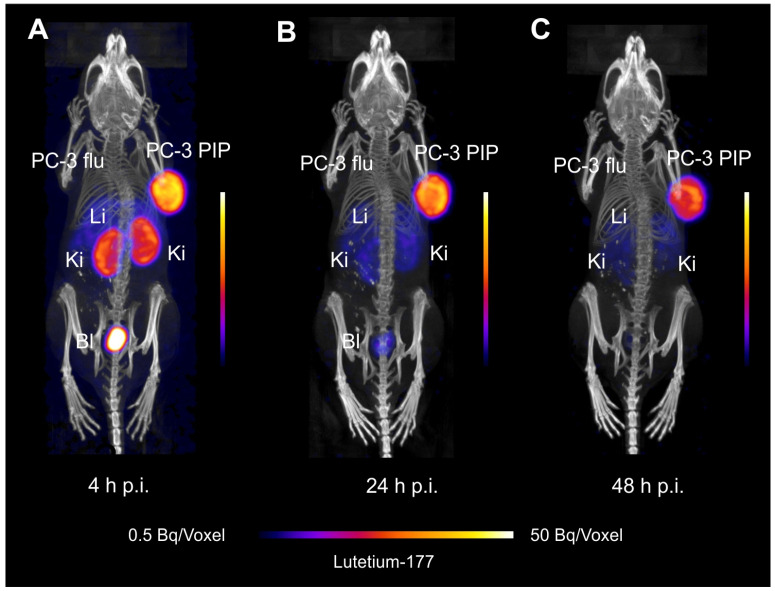
SPECT/CT images of a PC-3 PIP/flu tumor-bearing mouse injected with [^177^Lu]Lu-PSMA-TB-01. (**A**) Image acquired at 4 h p.i.; (**B**) image acquired at 24 h p.i., and (**C**) image acquired at 48 h p.i. (Li: liver, Ki: kidney, Bl: urinary bladder).

SPECT/CT imaging studies illustrated the high accumulation of [^177^Lu]Lu-PSMA-TB-01 in the PSMA-positive PC-3 PIP tumor xenograft and negligible uptake in the PSMA-negative PC-3 flu tumor (Figure 5). Activity was also seen in the kidney at 4 h p.i. (Figure 5A), which decreased over time while the radioligand was retained in the PC-3 PIP tumor xenograft (Figure 5B,C). The retention of activity in other organs and tissues was low and only observable at early timepoints, mainly in the liver. The tissue distribution profile visualized by SPECT/CT correlated well with the activity accumulation in organs and tissues determined in biodistribution studies at the respective timepoint (Supplementary Materials, Table S2).

## 4. Discussion

In this study, we explored the possibility of using a transthyretin-binding entity (TB-01) [17,22] to enhance the blood circulation of the resultant PSMA radioligand, [^177^Lu]Lu-PSMA-TB-01, as an alternative approach to the widely used albumin binder concept. The synthesis of PSMA-TB-01 was carried out using a well-established peptide synthesis methodology previously reported by Benešova et al. [14]. The final chemical conjugation step, as well as the subsequent cleavage from the resin and global deprotection, required, however, an extensive optimization of the reaction conditions to prevent the formation of byproducts that would have complicated the HPLC purification process.

In physiologic solution, [^177^Lu]Lu-PSMA-TB-01 was stable over 24 h (>99% intact radioligand). This was in contrast to the reported rapid radiolytic degradation of [^177^Lu]Lu-PSMA-617, which resulted in <2% intact radioligand after 24 h of incubation using the same experimental conditions [11]. The increased stability of [^177^Lu]Lu-PSMA-TB-01 was in agreement with the stabilizing effect of albumin binders, previously observed for [^177^Lu]Lu-PSMA-ALB-53 and [^177^Lu]Lu-PSMA-ALB-56, which were modified with the *p*-iodophenyl and *p*-tolyl entities, respectively [11]. The hydrophilic properties (logD value) remained in the same range for [^177^Lu]Lu-PSMA-TB-01 as was previously found for [^177^Lu]Lu-PSMA-617 [11]. The modification of the PSMA radioligand with TB-01 had no major influence on the PSMA-binding affinity (K_D_: 23 ± 1 nM), which was in the same range as for the parent compound [^177^Lu]Lu-PSMA-617 (K_D_: 13 ± 1 nM [12]). The derivatization with the transthyretin binder also did not affect the target-specific cell uptake, which remained high in the PSMA-positive PC-3 PIP tumor cells, while only negligible uptake was seen in PSMA-negative PC-3 flu cells.

The in vitro investigations revealed that the binding of [^177^Lu]Lu-PSMA-TB-01 to transthyretin was ~174-fold stronger relative to the binding to HSA. However, since HSA is present in human blood plasma (~700 µM) in an ~140-fold higher molar concentration than transthyretin (~5 µM), the bound fraction increased when using human blood plasma. In undiluted blood plasma, the fraction of [^177^Lu]Lu-PSMA-TB-01 bound to serum proteins was considerably higher (93 ± 1%) than for the parent compound [^177^Lu]Lu-PSMA-617 (59 ± 1% [12]). It was almost equal to the protein-bound fraction of the previously developed [^177^Lu]Lu-PSMA-ALB-56 (95 ± 1% [12]), which was modified with the *p*-tolyl-based albumin binding entity [11] and even slightly higher than for [^177^Lu]Lu-SibuDAB (88 ± 2%; [25]) modified with (*S*)-ibuprofen as an albumin-binding entity. It is also relevant to note that the serum-protein-bound fraction of [^177^Lu]Lu-PSMA-TB-01 was comparable in mouse and human blood plasma. Indeed, biodistribution data in mice demonstrated a substantially enhanced blood retention of [^177^Lu]Lu-PSMA-TB-01 compared to the fast excretion of [^177^Lu]Lu-PSMA-617, indicating that the transthyretin-binder concept is also valid in vivo. In line with the in vitro plasma protein binding, blood clearance of [^177^Lu]Lu-PSMA-TB-01 was considerably faster than it was for [^177^Lu]Lu-PSMA-ALB-56 (16 ± 1% IA/g at 4 h p.i.) [11] but somewhat slower than for [^177^Lu]Lu-SibuDAB (2.9 ± 1.9% IA/g at 4 h p.i.) [25].

As a result of the enhanced blood circulation of [^177^Lu]Lu-PSMA-TB-01, the accumulation in the tumor tissue was slower than seen with conventional PSMA radioligands such as [^177^Lu]Lu-PSMA-617. Over time, a higher uptake of activity was reached in PC-3 PIP xenografts (71 ± 10% IA/g at 24 h p.i.) after injection of [^177^Lu]Lu-PSMA-TB-01 than previously observed with [^177^Lu]Lu-PSMA-617 (37 ± 6% IA/g at 24 h p.i.) [11]. The tumor accumulation and retention of [^177^Lu]Lu-PSMA-TB-01 were comparable to the previous data obtained for [^177^Lu]Lu-PSMA-ALB-56 and [^177^Lu]Lu-SibuDAB [25]. The retention in the kidneys was higher for [^177^Lu]Lu-PSMA-TB-01 than for [^177^Lu]Lu-PSMA-617 as it was seen with virtually all PSMA radioligands modified with an albumin-binding entity [11,12,14,15,16]. SPECT/CT studies visualized the described tissue distribution profile of [^177^Lu]Lu-PSMA-TB-01 with a negligible accumulation of activity in the PC-3 flu tumor xenografts, which confirmed the PSMA-specific tumor uptake of the novel radioligand. Clearly, the enhanced circulation time of [^177^Lu]Lu-PSMA-TB-01 serves the therapy concept for which high tumor uptake and retention would be beneficial for the delivery of a sufficient radiation dose to eliminate metastases. On the other hand, PET imaging agents are preferentially characterized with fast pharmacokinetics resulting in rapid tumor uptake as is the case for the currently used [^68^Ga]Ga-PSMA-11 [7,28]. In this case, it is relevant to reach a high tumor-to-background contrast early after radioligand application to enable PET imaging shortly after injection into the patient [29].

A more in-depth investigation of the binding of [^177^Lu]Lu-PSMA-TB-01 to plasma proteins revealed that the protein-bound fraction of the radioligand in a solution of transthyretin was reduced by >60% in the presence of a 100-fold excess of thyroxine or TB-01. These results indicate a site-specific binding of [^177^Lu]Lu-PSMA-TB-01 to the thyroxine-binding pocket present on the interface of the subunits of the transthyretin tetramer [30]. The observation of a reduction in binding of the radioligand to transthyretin in the presence of a large excess of ibuprofen and warfarin is in line with the fact that ibuprofen and other non-steroidal anti-inflammatory drugs bind to transthyretin and stabilize the tetramer [30]. Blocking studies performed with a solution of HSA revealed a pronounced reduction in the bound fraction of [^177^Lu]Lu-PSMA-TB-01 in the presence of a 100-fold excess of warfarin or ibuprofen. This indicates the binding of [^177^Lu]Lu-PSMA-TB-01 to both Sudlow site I (warfarin-binding site) and Sudlow site II (ibuprofen-binding site) [31]. On the other hand, the binding of [^177^Lu]Lu-PSMA-TB-01 to HSA was not affected in the presence of thyroxine or TB-01, which is in line with the low HSA affinity of this class of compounds [32].

The concept of using a transthyretin-binding entity has also been investigated in combination with other therapeutic payloads, including peptides [17] and small-molecular-weight drug conjugates [20,21]. Aside from the improved in vivo half-life, previous studies have highlighted the potential use of a transthyretin-binder for the delivery of hydrophobic cytotoxic drugs to tumor tissue [21]. Similarly, we observed that the hydrophilic properties of the transthyretin binder (TB-01) enabled to maintain the hydrophilic properties of the resultant radioligand visible by comparable logD values of [^177^Lu]Lu-PSMA-TB-01 (logD: −3.4) and [^177^Lu]Lu-PSMA-617 (logD: −4.4 [14]), while the use of the *p*-iodophenyl and *p*-tolyl entities ([^177^Lu]Lu-PSMA-ALB-53/56, logD values: −2.7 to −2.9) commonly enhanced the lipophilicity of the resultant radioligands. The increased lipophilicity upon the modification with an albumin binder has also been observed using other tumor targeting agents, which resulted in higher background activity [33,34]. Hydrophilic properties of radiopharmaceuticals are commonly desirable to favor renal excretion and prevent background activity in the liver and intestinal tract. The availability of hydrophilic plasma protein binders, as is the case for TB-01, may be of relevance in view of the design of future radiopharmaceuticals, in particular when the radioligand backbones are already lipophilic [35,36,37].

## 5. Conclusions

Our findings with a TB-01-modified PSMA radioligand demonstrate for the first time the successful implementation of the transthyretin binder concept to enhance the circulation time of a small-molecule-based radioligand. The tumor uptake of [^177^Lu]Lu-PSMA-TB-01 was higher than that previously reported for [^177^Lu]Lu-PSMA-617 and comparable to values of other known PSMA radioligands modified with moderately affine albumin binders. This example may pave the way for future applications of this transthyretin binder in conjunction with other tumor-targeting agents, in particular when hydrophilic properties would be desirable.

## Data Availability

The data presented in this study are available on request from the corresponding author.

## Supplementary Materials

The following supporting information can be downloaded at: https://www.mdpi.com/article/10.3390/cancers16071262/s1. Scheme S1: Synthesis of resin-immobilized intermediate 1; Scheme S2: Multistep organic synthesis of MeTB-01; Figure S1: Radiochromatogram of [^177^Lu]Lu-PSMA-TB-01 labeled at a molar activity of 50 MBq/nmol; Table S1: Half-maximum binding (B50) and relative binding affinities of [^177^Lu]Lu-PSMA-TB-01 to various plasma proteins; Table S2: Biodistribution of [^177^Lu]Lu-PSMA-TB-01 in mice with PC-3 PIP/flu tumor xenografts.
