# Supplementary material for: Design and Preclinical Evaluation of a Novel Prostate-Specific Membrane Antigen Radioligand Modified with a Transthyretin Binder
Source: Cancers (Basel). 2024 Mar 23;16(7):1262. doi: 10.3390/cancers16071262 (PMC11011029; doi:10.3390/cancers16071262)
Supplement: Supplementary file 1 [file cancers-16-01262-s001.zip › cancers-2896755-supplementary.pdf]

SUPPLEMENTARY MATERIAL

Design and Preclinical Evaluation of a Novel Prostate-Specific Membrane Antigen Radioligand Modified with a Transthyretin Binder

Christian Vaccarin[1†], Ana Katrina Mapanao[1†], Luisa M. Deberle[1], Anna E. Becker[1], Francesca Borgna[1], Giovanni Marzaro[2], Roger Schibli[1,3], Cristina Müller[1,3*]

1. Center for Radiopharmaceutical Sciences ETH-PSI, Paul Scherrer Institute, 5232 Villigen-PSI, Switzerland
2. Department of Pharmaceutical and Pharmacological Sciences, University of Padua, I-35131 Padua, Italy
3. Department of Chemistry and Applied Biosciences, ETH Zurich, 8093 Zurich, Switzerland

[†] These authors contributed equally to this work.

E-Mail addresses:
christian.vaccarin@psi.ch; ana.mapanao@psi.ch; luisa.deberle@gmail.com; francesca.borgna.1@gmail.com; beckeranna.e@gmail.com; giovanni.marzaro@unipd.it; roger.schibli@psi.ch; cristina.mueller@psi.ch

***Correspondence to**:
Prof. Dr. Cristina Müller
Center for Radiopharmaceutical Sciences ETH-PSI
Paul Scherrer Institute
5232 Villigen-PSI
Switzerland
e-mail: cristina.mueller@psi.ch
phone: +41-56-310 44 54; fax: +41-56-310 28 49

1. Synthesis of the PSMA Ligand

Purpose: PSMA-TB-01 was synthesized to investigate the effect of the transthyretin binder in combination with a well-established PSMA ligand.

Methods & Results:

*Synthesis of the resin-immobilized intermediate **1**:* The resin-immobilized precursor (**1**) of the PSMA ligand was produced by standard fluorenylmethyloxycarbonyl (Fmoc)-based solid-phase peptide synthesis techniques involving an 11 steps reaction pathway accordingly to the methods previously reported by *Umbricht et al* (Scheme S1) [1].

Scheme S1. Synthesis of the resin-immobilized intermediate 1.

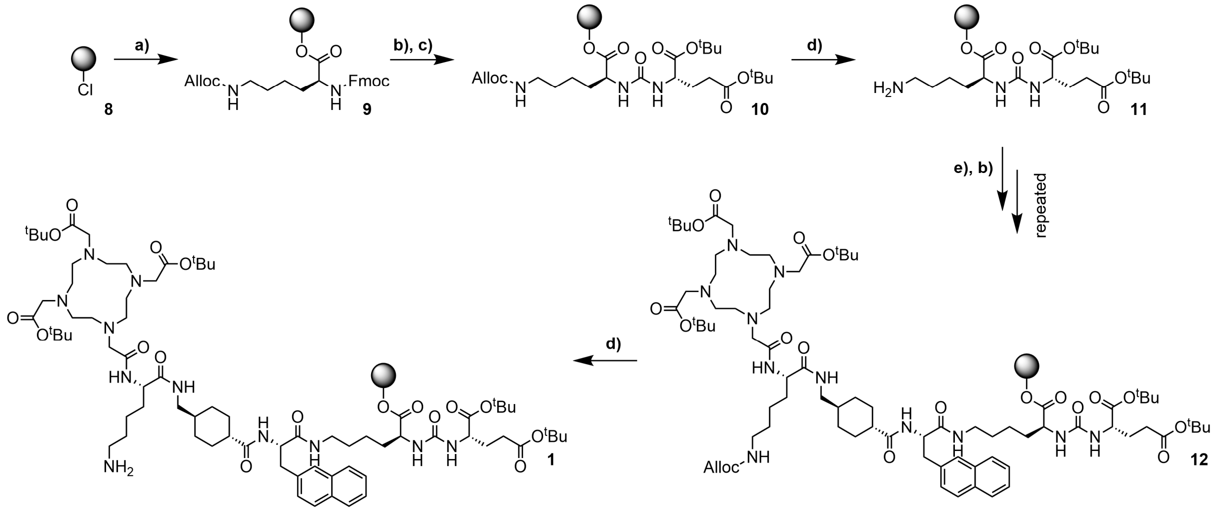

Reaction conditions: **a**) Fmoc-Lys(Alloc)-OH, DIPEA, DCM, RT, overnight; **b**) 50% piperidine in DMF, RT, 2 x 5 min; **c**) H-Glu(O^{t}Bu)-O^{t}Bu x HCl, triphosgene, DIPEA, DCM, 0°C to RT, overnight; **d**) Pd(PPh$_3$)$_4$, morpholine, DCM, RT, 1 h; **e**) Carboxylic acid derivative, DIPEA, DMF, RT, 1 h. Sequence of carboxylic acid coupled: Fmoc-2-NaI-OH, *N*-Fmoc-tranexamic acid, Fmoc-Lys(Alloc)-OH, DOTA-tri(^{t}Bu ester).

Briefly, 2-chlorotrityl chloride resin (**8**, 0.1 mmol) swelled in anhydrous dichloromethane for 45 min, then, *N*α-Fmoc-*N*ε-(4-allyloxycarbonyl)-l-lysine (Fmoc-Lys(Alloc)-OH, 54 mg, 0.12 mmol, 1.2 equiv) and *N*,*N*-diisopropylethylamine (DIPEA, 139 µL, 0.8 mmol, 8.0 equiv) were dissolved in dry dichloromethane (DCM, 3 mL), added to the resin, and stirred overnight (step **a**). Potentially unreacted 2-CTC resin was capped with a mixture of DCM, methanol (MeOH) and DIPEA (17:2:1, *v/v/v*) for 30 min. After each reaction step, unreacted reagents were removed from the resin by washing with DCM or dimethylformamide (DMF). Subsequently, the Fmoc protecting group was removed by shaking the resin in a mixture of DMF and piperidine (5:5, *v/v*) twice for 5 min (step **b**). In parallel, a solution of DIPEA (5 mL) in DCM (6 mL) was slowly added to an ice-cold solution of glutamic acid di-*tert*-butyl ester hydrochloride (H-Glu(O^{t}Bu)-O^{t}Bu x HCl, 295 mg, 1.0 mmol, 10.0 equiv) and triphosgene (98 mg, 0.33 mmol, 3.3 equiv) in DCM (3 mL) over 1 h followed by further stirring at room temperature (RT) for 2 h. At this stage, the resin-immobilized compound was added to the reaction mixture which was then stirred at RT overnight (step **c**). At reaction completion, the resin was collected by filtering through

a filter-containing 5 mL syringe and thoroughly washed with DCM and DMF. The *N*ε-Alloc protecting group present compound **10** was cleaved with tetrakis(triphenylphosphine)palladium(0) ($Pd(PPh_3)_4$, 35 mg, 0.03 mmol, 0.3 equiv) in the presence of morpholine (260 µL, 3.0 mmol, 30.0 equiv) in dry DCM within 1 h (step **d**). To remove residual palladium traces, the resin-immobilized compound was washed with 1% DIPEA in DMF (*v/v*) and then with a solution of sodium diethyldithiocarbamate (15 mg/mL) in DMF. Fmoc-3-(2-naphthyl)-L-alanine (Fmoc-2-Nal-OH, 175 mg, 0.4 mmol, 4.0 equiv), was activated for 2 min with O-(benzotriazol-1-yl)-*N*,*N*,*N'*,*N'*-tetramethyluronium-hexafluorophosphate (HBTU, 150 mg, 0.396 mmol, 3.96 equiv) in the presence of DIPEA (139 µL, 0.8 mmol, 8.0 equiv) in dry DMF before being added to the resin (**11**) and agitated for 1 h, after which the Fmoc protecting group was cleaved as described above. At this point, steps **e** and **b** were repeated with *N*-Fmoc-tranexamic acid (152 mg, 0.4 mmol, 4.0 equiv), Fmoc-Lys(Alloc)-OH (181 mg, 0.4 mmol, 4.0 equiv) and tri-*tert*-butyl 1,4,7,10-tetraaza-cyclododecane-1,4,7,10-tetraacetate (DOTA-tri(^{t}Bu ester), 259 mg, 0.4 mmol, 4.0 equiv). The cleavage of the remaining *N*ε-Alloc protecting group present on the resin (**12**) was performed as described above (step **d**) to obtain the desired resin-immobilized intermediate **1**. The chemical identity of the compound was confirmed by exposing a small portion of the resin (10 mg) to trifluoroacetic acid (TFA) followed by analysis of the supernatant by liquid chromatography–mass spectrometry (LC−MS). LC-MS for $C_{55}H_{83}N_{11}O_{17}$ $[M-H]^-$: calculated 1168.6, found 1169.0.

Multistep synthesis of MeTB-01: The intermediate MeTB-01 was produced by a convergent synthetic approach involving a 5-step reaction pathway (Scheme S2).

Scheme S2. Multistep organic synthesis of MeTB-01.

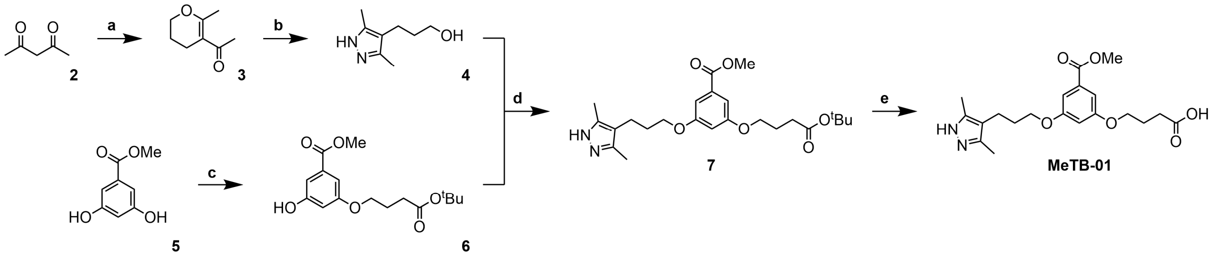

Reaction conditions: **a**) 1,3-dibromopropane, K_2CO_2, KI, acetone, reflux, 16 h, yield: 85%; **b**) hydrazine hydrate, EtOH, reflux, 16 h, yield: 73%; **c**) *tert*-butyl 4-bromobutanoate, K_2CO_2, KI, acetone, reflux, 16 h, yield: 34%; **d**) PPh_3, DIAD, THF, RT, 16 h, yield: 87%; **e**) DCM, TFA, RT, 30 min, yield: 99%.

Synthesis of 3,4-dihydro-6-methyl-2H-pyran-5-yl methyl ketone (3): A mixture of pentane-2,4-dione (**2**, 1.02 mL, 10.0 mmol, 1.0 equiv), K_2CO_3 (1.38 g, 10.0 mmol, 1.0 equiv), catalytic KI (10 mg) and 1,3-dibromopropane (1.33 mL, 13.0 mmol, 1.3 equiv) in acetone (10 mL) was heated to reflux for 16 h. The reaction mixture was monitored by thin layer chromatography (TLC, eluent: (EtOAc)/cyclohexane (CE), 2:8, *v*/*v*). After cooling, the insoluble material was removed by filtration and the filtrate was obtained as a clear solution which was concentrated under reduced pressure. The resulting residue was

purified by column chromatography using a mixture of EtOAc and CE (2:8, *v*/*v*) as eluent to afford compound **3** as a light-yellow oil. Yield: 85%. ^{1}H-NMR ($CDCl_3$-*d*): 4.04 (t, J = 5.2 Hz, 2H,), 2.40 – 2.36 (m, 2H), 2.22 (s, 3H), 2.21 (t, J = 1.4 Hz, 3H), 1.93 – 1.87 (m, 2H).

Synthesis of 3-(3,5-dimethyl-1H-pyrazol-4-yl)propan-1-ol (4). A solution of compound **3** (1.12 g, 8.0 mmol, 1.0 equiv) and hydrazine monohydrate 65% (0.74 mL, 9.6 mmol, 1.2 equiv) in ethanol (EtOH, 5 mL) was heated to reflux for 16 h. The reaction mixture was monitored by TLC (eluent: $CHCl_3$/MeOH, 9:1, *v*/*v*). After cooling, the solution was concentrated under reduced pressure and the residue was purified by column chromatography using a mixture of $CHCl_3$ and MeOH (95:5, *v*/*v*) as eluent. The residue was then taken up in DCM (20 mL), added of anhydrous Na_2SO_4 (5 g) and refluxed for 2 h. The suspension was filtered while still hot to remove the inorganic salt and the clear solution was dried under vacuum, yielding compound **4** as white crystals. Yield: 73%. ^{1}H-NMR (CD_3OD-d_4): 3.53 (t, J = 6.6 Hz, 2H), 2.44 (t, J = 7.4 Hz, 2H), 2.16 (s, 6H), 1.71 – 1.62 (m, 2H).

*Synthesis of methyl 3-(4-(tert-butoxy)-4-oxobutoxy)-5-hydroxybenzoate (**6**):* K_2CO_3 (4.14 g, 10.0 mmol, 1.0 equiv) was added to a solution of methyl-3,5-dihydroxybenzoate (**5**, 5.04 g, 30.0 mmol, 3.0 equiv) in acetone (60 mL) and stirred at RT for 30 min. KI (50 mg) and *tert*-butyl 4-bromobutanoate (6.69 g, 10.0 mmol, 1.0 equiv) were added and the reaction mixture was heated to reflux for 16 h. The reaction mixture was monitored by TLC (eluent: $CHCl_3$/MeOH, 9:1, *v*/*v*). After cooling, the insoluble material was removed by filtration and the clear solution was concentrated under reduced pressure. The resulting residue was purified by column chromatography using a mixture of EtOAc and CE (1:9, *v*/*v*) as eluent to afford compound **6** as white crystals. Yield: 34%. ^{1}H-NMR (CD_3OD-d_4): 7.02 (d, J = 2.2 Hz, 2H), 6.57 (t, J = 2.2 Hz, 1H), 3.99 (t, J = 6.2 Hz, 2H), 3.87 (s, 3H), 2.41 (t, J = 7.2 Hz, 2H), 2.06 – 1.99 (m, 2H), 1.45 (s, 9H).

Synthesis of Methyl 3-(4-(tert-butoxy)-4-oxobutoxy)-5-(3-(3,5-dimethyl-1H-pyrazol-4-yl)propoxy)-benzoate (7): Compound **4** (1.91 g, 6.2 mmol, 1.0 equiv), compound **6** (1.04 g, 6.8 mmol, 1.1 eqiuv) and triphenylphosphine (1.78 g, 6.8 mmol, 1.1 equiv) were dissolved in tetrahydrofuran (THF, 2.5 mL) and the mixture was sonicated for 2 min. Ice-cold diisopropyl azodicarboxylate (DIAD, 1.33 mL, 6.8 mmol, 1.1 equiv) was added dropwise to the ice-cold suspension over the course of 15 min, after which the reaction mixture was left stirring overnight at RT. The resulting mixture was monitored by TLC (eluent: EtOAc/CE, 4:6, *v*/*v*) and, after completion, the reaction was quenched with a saturated solution of $NaHCO_3$ (50 mL) and extracted with EtOAc (3 x 20 mL). The organic phase was dried with Na_2SO_4, filtered and concentrated under reduced pressure. The resulting residue was purified by column chromatography using a mixture of $CHCl_3$ and MeOH (97:3, *v*/*v*) as eluent to afford compound **7** as white crystals. Yield: 87%. ^{1}H-NMR ($CDCl_3$-*d*): 7.14 (d, J = 2.2 Hz, 2H), 6.61 (t, J = 2.2 Hz, 1H), 4.00 (t, J = 6.2 Hz, 2H), 3.90 (t, J = 6.0 Hz, 2H), 3.88 (s, 3H), 2.54 (t, J = 7.2 Hz, 2H), 2.41 (t, J = 7.2 Hz, 2H), 2.17 (s, 6H), 2.09 – 2.01 (m, 2H), 1.96 – 1.87 (m, 2H), 1.44 (s, 9H).

*Synthesis of 4-(3-(3-(3,5-dimethyl-1H-pyrazol-4-yl)propoxy)-5-(methoxycarbonyl)phenoxy)butanoic acid (**MeTB-01**):* Compound **7** (1.01 g, 2.3 mmol, 1.0 equiv) was dissolved in DCM (6 mL) and TFA (6 mL) was added. The solution was stirred at RT for 30 min and monitored by TLC (eluent: $CHCl_3$/MeOH, 9:1, *v*/*v*). After completion, the solvents were evaporated under reduced pressure, affording MeTB-01 as white crystals. Yield: 99%. ^{1}H-NMR (CD_3OD-*d_4*): 7.15 (dd, J = 2.2, 1.2 Hz, 1H), 7.11 (dd, J = 2.2, 1.2 Hz, 1H), 6.71 (t, J = 2.2 Hz, 1H), 4.04 (t, J = 6.2 Hz, 2H), 3.97 (t, J = 6.0 Hz, 2H), 3.88 (s, 3H), 2.69 (t, J = 7.2 Hz, 2H), 2.49 (t, J = 7.2 Hz, 2H), 2.29 (s, 6H), 2.11 – 2.03 (m, 2H), 2.02 – 1.95 (m, 2H). HRMS for $C_{20}H_{25}N_2O_6$ $[M-H]^-$: calculated 389.1718, found 389.1695.

Synthesis of PSMA-TB-01: The synthesis scheme to prepare PSMA-TB-01 is depicted in the main article (Scheme 1). The resin-immobilized compound **1** (0.1 mmol) was swelled in anhydrous DCM for 45 min and subsequently conditioned with dry DMF. The carboxylic function of MeTB-01 (156 mg, 0.4 mmol, 4.0) was activated with HBTU (150 mg, 3.96 equiv, 0.396 mmol) in the presence of DIPEA (139 µL, 8.0 equiv, 0.8 mmol) in anhydrous DMF for 2 min. The resulting activated ester was coupled to the primary amine of precursor **1**. This reaction was performed two times for 1 h each before proceeding with the next reaction step to ensure a complete conversion of the resin-immobilized compound **2**. Cleavage from the resin was performed by agitating the resin in a solution of hexafluoroisopropanol in DCM (1:4; *v*/*v*) twice for 30 min. Removal of the ^{t}Bu protecting groups was accomplished by stirring the fully protected product in 50% TFA in DCM containing 5% triisopropyl silane (TIPS) and 5% Milli-Q water for 4.5 h. After evaporation of the solvents, the residual methyl ester protecting group was hydrolyzed by stirring in a LiOH solution (0.5 M, in a mixture of Milli-Q water/acetonitrile (MeCN), 1:1; *v*/*v*) for 1 h followed by the addition of a HCl solution (1 M) to reach pH 7. The purification of the compound was performed using HPLC with a Merck-Hitachi LaChrom HPLC system equipped with a D-7000 interface, a L-7200 autosampler, a L-7400 UV detector (λ = 254 nm), a L-7100 pump and a semi-preparative reversed-phase C18 column (5 µm, 10×150 mm, Sunfire™, Waters, Milford, US-MA). A linear gradient of 95–65% Milli-Q water with 0.1% TFA and 5–35% MeCN was applied over 20 min at a flow rate of 4 mL/min to enable separation of the desired product. The pure product fractions (t_R = 17.3 – 18.1 min) were collected, frozen in liquid nitrogen and lyophilized for ≥24 h. The chemical purity of the final compound was determined by LC-MS analysis performed on an Acquity SQD2 LC-MS system (Waters, Milford, USA) equipped with a reversed-phase C18 column (Acquity UPLC BEH, 1.7 µm, 2.1 x 50 mm, Waters, Milford, USA) by allying a linear gradient of Milli-Q water containing 0.1% formic acid (98–2%) and MeCN containing 0.1% formic acid (2–98%) over 4 min at a flow rate of 0.5 mL/min. The eluted compound was detected by determining its absorbance at λ = 254 nm. The chemical identity of the final compound was confirmed using high-resolution mass spectrometry. HRMS for $C_{74}H_{105}N_{13}O_{22}$ $[M+2H]^{2+}$: calculated 697.3556, found 697.3563.

2. Radiolabeling of PSMA-TB-01

Purpose: PSMA-TB-01 was labeled with lutetium-177 for *in vitro* and *in vivo* experiments.

Methods: PSMA-TB-01 was dissolved in Milli-Q water containing 25% DMSO to obtain a ligand concentration of 1 mM. The radiolabeling was performed at 50 MBq/nmol using lutetium-177 (no-carrier-added; in 0.04 M HCl; ITM Medical Isotopes, GmbH, Germany) at pH ~4.5 in a 1:5 (*v/v*) mixture of sodium acetate (0.5 M) and HCl (0.05 M). The reaction mixture was incubated for 10 min at 95 °C. Quality control was performed using a sample of the reaction mixture diluted in Milli-Q water containing sodium diethylenetriamine pentaacetic acid (Na_5-DTPA, 50 µM) using a reversed-phase C18 column (Xterra^{TM} MS, C18, 5 µm, 150 x 4.6 mm; Waters) connected to a Merck Hitachi LaChrom HPLC system, equipped with a D-7000 interface, a L-7200 autosampler, a radioactivity detector (LB 506 B; Berthold) and a L-7100 pump. A linear gradient of 95–20% Milli-Q water with 0.1% TFA and 5–80% MeCN was applied over 15 min at a flow rate of 1 mL/min [1,2].

Results: [^{177}Lu]Lu-PSMA-TB-01 was prepared at 50 MBq/nmol with a radiochemical purity >98% (Figure S1). This molar activity was the highest needed for the subsequent *in vitro* and *in vivo* studies. The retention time of [^{177}Lu]Lu-PSMA-TB-01 was relatively short (t_R = 9.4 min) indicating that the hydrophilic character of the ligand due to the transthyretin binder containing a free carboxylic acid group. Unreacted lutetium-177 would appear as [^{177}Lu]Lu-DTPA after ~3 min.

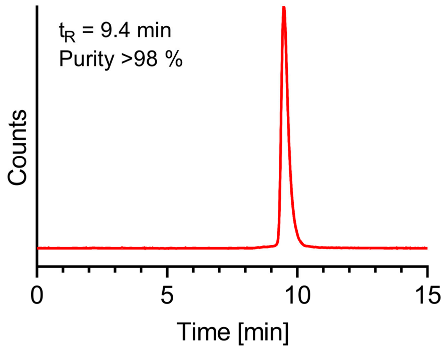

Figure S1. Radiochromatogram of [^{177}Lu]Lu-PSMA-TB-01 labeled at a molar activity of 50 MBq/nmol. The retention time (t_R) and radiochemical purity are indicated in the figure.

3. Radiolytic Stability of the PSMA Radioligand

Purpose: The radiolytic stability [^{177}Lu]Lu-PSMA-TB-01 was determined at an activity concentration representative of radiopharmaceutical formulations for clinical applications.

Methods: After preparation of [^{177}Lu]Lu-PSMA-TB-01 (50 MBq/nmol), quality control was performed using HPLC (t = 0, radiochemical purity >98% set as t_0 = 100%). The labeling solution (~60 μL) was diluted with PBS to 150 MBq in 300 μL. The radioligand dilution was incubated at RT and radiolytic degradation was quantified by HPLC analysis after 4 h and 24 h incubation period. The HPLC chromatograms were analyzed by integrating the peaks representing the radiolabeled product, the released lutetium-177 and degradation products of unknown structure. A quantitative assessment was performed by expressing the peak area of the intact product as percentage of the sum of integrated peak areas of the entire chromatogram and set into relation to the original value determined at t_0 which was set as 100%

Results: [^{177}Lu]Lu-PSMA-TB-01 was completely stable (>99% intact) over a 24-h incubation period.

4. *n*-Octanol/PBS Distribution Coefficient

Purpose: The distribution coefficient (logD value) was determined as a measure of the lipophilic/hydrophilic character of [^{177}Lu]Lu-PSMA-TB-01.

Methods: The logD value of [^{177}Lu]Lu-PSMA-TB-01 (50 MBq/nmol) was determined by the shake flask method as previously reported [1]. An aliquot of [^{177}Lu]Lu-PSMA-TB-01 (0.5 MBq; 10 pmol, 25 μL) was added to a vial containing a mixture of PBS (pH 7.4, 1475 μL) and *n*-octanol (1500 μL). After vortexing vigorously for 1 min, the vials were centrifuged (560 rcf; 6 min) to obtain phase separation. The quantity of activity in the organic and aqueous phases was determined in a γ-counter (Perkin Elmer, Wallac Wizard 1480). The distribution coefficients were expressed as the logarithm of the ratio of counts per minute (cpm) measured in the *n*-octanol phase to the cpm measured in the PBS phase. Three experiments were performed in quintuplicate and the value was presented as the average (± SD) of the three values.

Results: The results are reported in the main article.

5. Cell Uptake and Internalization

Purpose: The uptake and internalization of [^{177}Lu]Lu-PSMA-TB-01 was determined using PC-3 PIP and PC-3 flu tumor cells.

Methods: Uptake and internalization of [^{177}Lu]Lu-PSMA-TB-01 was investigated in PC-3 PIP and PC-3 flu cells similar to the procedure reported by Benešová *et al.* [2]. PC-3 PIP or PC-3 flu cells were seeded in 12-well plates (~3×10^5 cells in 2 mL standard growth medium/well) allowing adhesion and growth overnight. [^{177}Lu]Lu-PSMA-TB-01 (50 MBq/nmol) was diluted to 1.5 MBq/mL in saline containing 0.05% BSA. The tumor cells were rinsed once with PBS prior to the addition of RPMI-1640 cell culture medium (975 µL/well) and the radioligand (37.5 kBq, 0.75 pmol, 25 µL per well). The final BSA concentration in the well plates (0.00125%) did not impact the radioligand's behavior, but was necessary to prevent adherence of the radioligands to the material of well plates [2]. The well-plates were incubated for 2 h and 4 h at 37 °C and 5% CO_2. To determine the uptake of the radioligand, the cells were rinsed three times with ice-cold PBS. The internalized fraction was determined in cells, which were rinsed with ice-cold PBS and incubated with acidic stripping buffer (0.05 M glycine buffer in 100 mM NaCl, pH 2.8) for 10 min followed by an additional washing step with ice-cold PBS. After lysis of the cells with NaOH (1 M, 1 mL) and transfer into ria tubes, the samples were counted in a γ-counter (PerkinElmer, Wallac Wizard 1480). The protein concentration was determined for each sample using a Micro BCA Protein Assay kit (Pierce, Thermo Scientific) in order to standardize the measured activity to the amounts of proteins in each well. The experiments were performed three times in triplicate.

Results: The results are reported in the main article.

6. Determination of PSMA-Binding Affinity (K_D Value)

Purpose: The PSMA affinity (K_D values) of [^{177}Lu]Lu-PSMA-TB-01 was determined to investigate whether it was affected by the modification with the transthyretin-binding entity.

Methods: The PC-3 PIP cells were incubated with various concentrations (1–2000 nM) of the [^{177}Lu]Lu-PSMA-TB-01 (5 MBq/nmol). Non-specific binding was determined by co-incubation of the tumor cells with 2-phosphonomethyl pentanedioic acid (2-PMPA, 200 µM) to selectively block PSMA on the cell surface. After lysing the cells (NaOH, 1 M, 600 µL) and transferring to ria tubes, the samples were counted for activity in a γ-counter (Wallac Wizard 1480, PerkinElmer). The K_D value was determined by plotting the values of the specific binding (total binding minus unspecific binding determined with blocking agent) against the molar concentration of the added PSMA ligand. Nonlinear regression analysis was performed using GraphPad Prism software (version 8) to obtain the K_D value from one curve of 4 independent experiments performed in triplicate. The K_D values were reported as the average ± standard deviation (SD).

Results: The result is reported in the main article.

7. Affinity Determination of the Radioligand to Plasma Proteins

***Purpose*:** The relative affinities of [^{177}Lu]Lu-PSMA-TB-01 to transthyretin, HSA and proteins in human blood plasma were investigated.

Methods: The preparation of transthyretin and HSA solutions are described in the main article. To calculate the protein-to-radioligand molar concentration range, it was assumed that the transthyretin concentration in undiluted human blood plasma is 5 μM [3,4]. The ultrafiltration procedure was described in the main article and was performed according to a previously published protocol [5]. The percentage of protein-bound [^{177}Lu]Lu-PSMA-TB-01 was plotted against the protein-to-radioligand molar concentration ratios in logarithmic scale. A non-linear regression curve (specific binding with Hill slope, B_{max}= 100%) was fitted to the data points using GraphPad Prism (version 8) to obtain the half-maximum binding (B_{50}) values. Relative binding affinities of the radioligand to plasma proteins of human blood plasma and human serum albumin were calculated by taking the ratio of the inverse B_{50} value set in relation to the inverse B_{50} value obtained for transthyretin, which was set as 1.0.

Results: The results are reported in the main article. The B_{50} values and relative binding affinities are summarized in Table S1.

Table S1. Half maximum binding (B_{50}) and relative binding affinities of [^{177}Lu]Lu-PSMA-TB-01 to various plasma proteins

	Transthyretin	Human serum albumin	Human blood plasma*
B_{50}	14.9	2594	3.87
Relative binding	1.0	0.006	3.9

*Based on reported concentration of 5 μM transthyretin in human blood plasma.

8. Blocking of the Binding of [^{177}Lu]Lu-PSMA-TB-01 to Plasma Proteins

Purpose: The affinity of the radioligand to human transthyretin, HSA and proteins in human blood plasma was identified in the presence of competing ligands that are known to bind to transthyretin (thyroxine and TB-01) or HSA (warfarin and ibuprofen).

Methods: In order to identify whether the radioligand will bind to the thyroxine-binding pocket of transthyretin, a competition assay was performed in the presence of 100-fold excess thyroxine or TB-01. Thyroxine has a limited solubility in PBS and was, therefore, prepared at a stock concentration of 100 μM. TB-01 was similarly diluted to a concentration of 100 μM in PBS. Prior to the addition of radioligand, 75 μL of the thyroxine or TB-01 stock solutions were pre-incubated with transthyretin (75 μL of 1 μM solution) for 15 min at 37 °C. The radioligand (0.3 MBq, 6 pmol, 15 μL) was added and the sample was incubated for 30 min at 37 °C. The amount of protein-bound radioligand was determined following the ultrafiltration assay described in the main article and according to a previously published protocol [5]. Blocking experiments using the same concentrations of thyroxine and TB-01 were also performed in HSA (final HSA concentration: 70 μM) and diluted human plasma (final transthyretin concentration ~0.5 μM).
The binding of [^{177}Lu]Lu-PSMA-TB-01 to Sudlow's site I and Sudlow's site II were also investigated through competition assays using 100-fold excess concentration of warfarin (marker for Sudlow's site I) or ibuprofen (marker for Sudlow's site II). Stock solutions of 14 mM warfarin and ibuprofen were prepared in PBS. Prior to the addition of radioligand, 75 μL of warfarin or ibuprofen were pre-incubated with HSA (75 μL of 140 μM solution) for 15 min at 37 °C. The radioligand (0.3 MBq, 6 pmol, 15 μL) was added and the sample was incubated for 30 min at 37 °C. The amount of protein-bound radioligand was determined following the ultrafiltration assay described in the main article and according to a previously published protocol [5]. Blocking experiments using the same concentrations of warfarin and ibuprofen were also performed with transthyretin (final transthyretin concentration: 0.5 μM) and diluted human plasma (final transthyretin concentration ~0.5 μM).
All blocking experiments were performed in parallel with control experiments performed with only [^{177}Lu]Lu-PSMA-TB-01 incubated in dilutions of transthyretin (0.5 μM), HSA (70 μM) or human plasma (transthyretin concentration: 0.5 μM) without blocking agent. The protein-binding of these experiments was set as 100%.

Results: The results are described in the main article.

9. Biodistribution Studies

Purpose: Biodistribution studies were performed in PC-3 PIP/flu tumor-bearing BALB/c nude mice to investigate the tissue distribution profile of [^{177}Lu]Lu-PSMA-TB-01 at different timepoints.

Methods: The methods are described in detail in the main article.

Results: The results are described in the main article and summarized in Table S2.

Table S2. Biodistribution of [^{177}Lu]Lu-PSMA-TB-01 in mice with PC-3 PIP/flu tumor xenografts. Decay-corrected data of accumulated activity are shown as % IA/g tissue, representing the average ± SD.

	**[^{177}Lu]Lu-PSMA-TB-01**			
	**1 h p.i.**	**4 h p.i.**	**24 h p.i.**	**96 h p.i.**
	n=4	*n*=4	*n*=4	*n*=4
	[% IA/g]	[% IA/g]	[% IA/g]	[% IA/g]
Blood	16 ± 1	5.5 ± 2.0	0.82 ± 0.13	0.13 ± 0.03
Heart	5.7 ± 0.8	2.2 ± 0.7	0.58 ± 0.04	0.17 ± 0.02
Lung	12 ± 2	4.3 ± 1.6	0.88 ± 0.20	0.21 ± 0.04
Spleen	4.3 ± 0.2	3.3 ± 0.8	1.5 ± 0.3	0.86 ± 0.19
Kidneys	56 ± 5	47 ± 6	18 ± 3	4.0 ± 0.6
Stomach	2.0 ± 0.2	0.87 ± 0.30	0.39 ± 0.08	< 0.1
Intestine	2.0 ± 0.5	1.2 ± 0.6	0.33 ± 0.06	< 0.1
Liver	4.0 ± 0.6	3.5 ± 1.0	1.6 ± 0.3	0.66 ± 0.08
Muscle	1.7 ± 0.2	0.70 ± 0.26	0.23 ± 0.06	< 0.1
Bone	2.1 ± 0.2	0.94 ± 0.33	0.35 ± 0.06	0.12 ± 0.02
Salivary glands	3.5 ± 0.1	1.8 ± 0.5	0.65 ± 0.12	0.22 ± 0.03
Lacrimal glands	5.4 ± 0.8	2.6 ± 0.3	1.2 ± 0.1	0.54 ± 0.16
PC-3 PIP tumor	37 ± 4	69 ± 13	72 ± 11	31 ± 8
PC-3 flu tumor	3.1 ± 0.3	1.5 ± 0.4	0.81 ± 0.12	0.23 ± 0.04
	**PC-3 PIP tumor-to-background ratios**			
Tumor-to-blood	2.3 ± 0.1	13 ± 3	88 ± 5	234 ± 15
Tumor-to-kidney	0.68 ± 0.12	1.5 ± 0.1	4.0 ± 0.2	7.6 ± 1.7
Tumor-to-liver	9.2 ± 0.7	20 ± 3	44 ± 4	48 ± 15

References

1. Umbricht, C.A.; Benesova, M.; Schibli, R.; Müller, C. Preclinical development of novel PSMA-targeting radioligands: modulation of albumin-binding properties to improve prostate cancer therapy. *Mol Pharm* **2018**, *15*, 2297-2306, doi:10.1021/acs.molpharmaceut.8b00152.
2. Benesova, M.; Umbricht, C.A.; Schibli, R.; Müller, C. Albumin-binding PSMA ligands: optimization of the tissue distribution profile. *Mol Pharm* **2018**, *15*, 934-946, doi:10.1021/acs.molpharmaceut.7b00877.
3. Buxbaum, J.N.; Reixach, N. Transthyretin: the servant of many masters. *Cell Mol Life Sci* **2009**, *66*, 3095-3101, doi:10.1007/s00018-009-0109-0.
4. Penchala, S.C.; Miller, M.R.; Pal, A.; Dong, J.; Madadi, N.R.; Xie, J.; Joo, H.; Tsai, J.; Batoon, P.; Samoshin, V.; et al. A biomimetic approach for enhancing the in vivo half-life of peptides. *Nat Chem Biol* **2015**, *11*, 793-798, doi:10.1038/nchembio.1907.
5. Busslinger, S.D.; Becker, A.E.; Vaccarin, C.; Deberle, L.M.; Renz, M.L.; Groehn, V.; Schibli, R.; Müller, C. Investigations using albumin binders to modify the tissue distribution profile of radiopharmaceuticals exemplified with folate radioconjugates. *Cancers (Basel)* **2023**, *15*, doi:10.3390/cancers15174259.
